# SARS-CoV-2 N Protein Hijacks the m^6^A Reader YTHDF2 to Suppress Antiviral Gene Expression

**DOI:** 10.3390/v18050496

**Published:** 2026-04-24

**Authors:** Peihan Wu, Shuai Wang, Xu Li

**Affiliations:** 1 Westlake Institute for Advanced Study, Fudan University, Shanghai 200032, China; wupeihan@westlake.edu.cn; 2 Key Laboratory of Integrated Oncology and Intelligent Medicine of Zhejiang Province, Hangzhou First People’s Hospital, Hangzhou 310006, China; 3 Center for Infectious Disease Research, Westlake Laboratory of Life Sciences and Biomedicine, Hangzhou 310024, China

**Keywords:** SARS-CoV-2, nucleocapsid protein, YTHDF2, m^6^A RNA methylation, innate immunity, host–virus interaction, interferon-stimulated genes, immune evasion

## Abstract

The m^6^A RNA methylation pathway plays a critical role in host antiviral defense. Host cells employ m^6^A readers such as YTHDF2 to regulate viral RNA fate through diverse mechanisms, including degradation, translational control, and immune recognition. However, we found that YTHDF2 is essential for SARS-CoV-2 replication, suggesting that a virus may exploit this host machinery to its advantage. Through integrative RNA-proteome analysis, we identified the SARS-CoV-2 nucleocapsid (N) transcript as the most heavily m^6^A-modified viral transcript and a direct interactor of YTHDF2. The N protein forms a complex with YTHDF2 in the cytoplasm and redirects this host RNA decay machinery toward host antiviral transcripts. N suppresses ISG15, IFIT1, MX1 and pro-inflammatory cytokines in a largely YTHDF2-dependent manner, an effect that is lost in YTHDF2-knockout cells. These findings reveal a viral immune evasion strategy wherein a viral protein actively hijacks an m^6^A reader to silence antiviral gene expression, establishing the N-YTHDF2 axis as a therapeutic target against SARS-CoV-2 and other coronaviruses.

## 1. Introduction

The coronavirus disease 2019 (COVID-19) pandemic, caused by severe acute respiratory syndrome coronavirus 2 (SARS-CoV-2), has posed an unprecedented global public health threat since its emergence in late 2019, with sustained transmission and the continuous emergence of variants of concern (VOCs) [1,2]. Despite the development of multiple vaccines and antiviral agents (e.g., Paxlovid), the virus’s remarkable capacity for immune evasion and rapid mutation continues to limit the efficacy of existing countermeasures [3]. This highlights the urgent need to dissect the fundamental molecular mechanisms underlying SARS-CoV-2-host interactions, particularly viral immune evasion strategies, to identify novel and durable antiviral targets.

The SARS-CoV-2 genome is a single-stranded, positive-sense RNA of ~30 kb, encoding four structural proteins (spike (S), envelope (E), membrane (M), and nucleocapsid (N)), 16 non-structural proteins (Nsp1–Nsp16), and several accessory proteins (ORF3a, ORF6, ORF7a, etc.) [4]. While the S protein has been the primary focus of vaccine and drug development due to its role in viral entry and as a major neutralizing antibody target [5,6], the N protein is increasingly recognized as a multifunctional virulence factor with critical roles in viral replication and host immune modulation [7]. As the most conserved structural protein among coronaviruses [8], the N protein mediates the packaging of the viral RNA genome into helical ribonucleoprotein (RNP) complexes, and regulates viral transcription and assembly through liquid–liquid phase separation (LLPS) [9]. Beyond its structural functions, accumulating evidence indicates that the N protein actively subverts the host innate immune response, the first line of defense against viral infection, by interfering with interferon (IFN) signaling and pro-inflammatory cytokine production [10,11].

Post-transcriptional RNA modifications, particularly N^6^-methyladenosine (m^6^A), the most abundant internal modification of eukaryotic mRNA, have emerged as a key regulatory layer in host–virus interactions [12,13]. The m^6^A modification is dynamically regulated by a set of core proteins: “writers” (e.g., METTL3/METTL14 complex) that install the modification, “erasers” (e.g., FTO, ALKBH5) that remove it, and “readers” (e.g., YTHDF1/2/3, YTHDC1/2) that recognize and interpret the m^6^A mark to regulate mRNA stability, translation, and subcellular localization [14,15]. The m^6^A pathway acts as a critical battleground between host and virus: the host can exploit m^6^A to target viral RNA for degradation and activate innate immunity, while viruses can hijack the m^6^A machinery to promote their own replication and evade immune surveillance [16,17]. For SARS-CoV-2, viral RNA is extensively modified by m^6^A [18,19], and this modification has been shown to have dual effects—either restricting viral replication by enhancing innate immune recognition or promoting viral infection by regulating viral RNA translation [18,20].

YTHDF2, a well-characterized m^6^A reader, exerts its primary function by recognizing m^6^A-modified mRNAs and recruiting the RNA decay machinery to promote their degradation [21,22]. Its role in viral infections is complex and context-dependent, acting as either a pro-viral or antiviral factor depending on the virus type. For example, YTHDF2 promotes influenza A virus (IAV) and porcine epidemic diarrhea virus (PEDV) replication by degrading antiviral genes (e.g., IFN-β, TRAF3) and suppressing the IFN response [23,24]; in contrast, YTHDF2 restricts Epstein–Barr virus (EBV) replication by targeting viral transcripts for degradation [25]. While previous studies have implicated the m^6^A pathway in SARS-CoV-2 infection [18,19], the specific role of YTHDF2, and its potential interactions with viral proteins, remain largely uncharacterized.

In this study, we report that the SARS-CoV-2 N, directly interacts with YTHDF2 and hijacks this host m^6^A reader to suppress the expression of key antiviral ISGs. We demonstrate that this immunosuppressive effect is largely YTHDF2-dependent, and that the N-YTHDF2 interaction represents a novel axis of viral immune evasion that does not rely on viral RNA stabilization, but rather on the usurpation of host RNA decay machinery to silence antiviral gene expression. Our findings uncover a new molecular mechanism underlying SARS-CoV-2 pathogenesis and identify the N-YTHDF2 interaction as a promising novel target for antiviral therapy development.

## 2. Materials and Methods

### 2.1. Constructs

The human YTHDF2 gene was amplified from HEK293T cell cDNA by PCR and cloned into the pDONR201 entry vector (Invitrogen, Carlsbad, CA, USA). The SARS-CoV-2 N protein gene was a kind gift from Dr. Peihui Wang. Gateway LR recombination was used to transfer YTHDF2 and N protein genes into Gateway-compatible destination vectors for the expression of SFB (S-tag, Flag-tag, Streptavidin-binding peptide) triple-tagged viral proteins and Myc-tagged host proteins. Deletion mutants of the N protein were generated by site-directed mutagenesis and verified by sequencing. For CRISPR-Cas9-mediated knockout, single-guide RNAs (sgRNAs) targeting YTHDF2 (5′-CTTACTTGAGTCCACAGGCA-3′) were synthesized and cloned into the lentiCRISPRv2 vector (Addgene, #52961). All plasmids were verified by sequencing before use.

### 2.2. Cell Culture and Transfection

HEK293T (ATCC, CRL-3216) and H1299 (ATCC, CRL-5803) cells were purchased from the American Type Culture Collection (ATCC, Manassas, VA, USA) and cultured in an incubator at 37 °C with 5% CO_2_. HEK293T cells were maintained in Dulbecco’s Modified Eagle’s Medium (DMEM, Yuanpei, Shanghai, China) supplemented with 10% fetal bovine serum (FBS, Gibco, Thermo Fisher Scientific, Waltham, MA, USA) and 1% penicillin-streptomycin (Yuanpei). H1299 cells were cultured in RPMI-1640 medium (Yuanpei) with the same supplements. All cell lines were routinely tested for mycoplasma contamination using the MycoAlert Mycoplasma Detection Kit (Lonza, Basel, Switzerland) and confirmed to be mycoplasma-free.

For the establishment of H1299 cells stably expressing SFB-tagged N protein (cSFB-N), cells were transfected with the cSFB-N plasmid using polyethyleneimine (PEI, Polysciences, Warrington, PA, USA). Transfected cells were selected with 2 μg/mL puromycin (Sangon Biotech, Shanghai, China) for 14 days, and single clones were isolated and verified by Western blotting. For transient transfections, cells were seeded in 6-well or 24-well plates and transfected with plasmids using PEI according to the manufacturers’ protocol.

### 2.3. Generation of CRISPR-Induced KOs

YTHDF2-knockout H1299 cell lines were generated using the CRISPR-Cas9 system. The lentiCRISPRv2-YTHDF2 sgRNA plasmid was co-transfected with the packaging plasmids pMD2.G (Addgene, #12259, Watertown, MA, USA) and psPAX2 (Addgene, #12260) into HEK293T cells to produce lentivirus. Viral supernatants were collected at 48 h post-transfection, filtered through a 0.45 μm filter, and used to infect H1299 cells. Infected cells were selected with 2 μg/mL puromycin for 14 days, and single clones were expanded. The knockout efficiency was verified by Western blotting with a YTHDF2-specific antibody, and clones with complete loss of YTHDF2 expression were used for subsequent experiments.

### 2.4. siRNA Transfection

Transient siRNA transfection was performed using the siRNA-mate plus reagent (GenePharma, Shanghai, China) according to the manufacturer’s instructions. Cells were seeded in 24-well plates or 96-well plates one day before transfection and cultured overnight at 37 °C in a humidified incubator with 5% CO_2_ until they reached approximately 60% confluence. For 24-well plates, 15 pmol of siRNA was diluted in 8.5 μL of GenePharma Buffer. When a 20 μM siRNA stock was used, 0.75 μL of siRNA solution was added. Then, 1.5 μL of siRNA-mate plus transfection reagent (GenePharma, Shanghai, China) was added directly to the diluted siRNA and mixed thoroughly by gentle pipetting to form the siRNA/transfection reagent complex. The complex was immediately added dropwise to the cells, and the plate was gently rocked to ensure even distribution. Cells were then returned to the incubator for further culture. Gene silencing efficiency was assessed by RT-qPCR at 48 h after transfection. siRNAs targeting YTHDF2, YTHDF2-siRNA-1#, 5′-GCCAUGUCAGAUUCCUACUTT-3′ (forward) and 5′-AGUAGGAAUCUGACAUGGCTT-3′ (reverse); YTHDF2-siRNA-2#, 5′-GGGAAAUAACAGUUCUCAGTT-3′ (forward), 5′-CUGAGAACUGUUAUUUCCCTT-3′ (reverse); YTHDF2-siRNA-3#, 5′-AUCGUGGCUUCCAAUAGUUTT-3′ (forward), 5′-AACUAUUGGAAGCCACGAUTT-3′ (reverse), Negative control -siRNA, 5′-UUCUCCGAACGUGUCACGUTT-3′(forward), 5′-ACGUGACACGUUCGGAGAATT-3′ (reverse).

### 2.5. Western Blotting and Co-IP

For Western blotting analysis, cells were lysed in NETN buffer (20 mM Tris-HCl, pH 8.0; 1 mM EDTA; 100 mM NaCl; 0.5% NP-40) and incubated on a rotator at 4 °C for 30 min. The lysates were clarified by centrifugation at 12,000 rpm for 15 min at 4 °C. Protein concentrations were determined using a bicinchoninic acid (BCA) assay kit (Thermo Fisher Scientific, Waltham, MA, USA), and all samples were adjusted to equal protein concentrations. Subsequently, 2× loading buffer (Beyotime, Shanghai, China) was added, and samples were denatured at 100 °C for 10 min. Proteins were separated by SDS-PAGE using SurePAGE™ Bis-Tris gels (4–20%, 10 × 8 cm, 15 wells; GenScript, Nanjing, China) and transferred onto 0.45 μm PVDF membranes (Millipore, Burlington, MA, USA). The membranes were incubated with the indicated primary antibodies at 4 °C overnight or at room temperature for 2 h, followed by incubation with secondary antibodies (1:5000; GenScript, Nanjing, China) for 1 h at room temperature. Protein bands were detected using an enhanced chemiluminescence (ECL) kit (GenScript, Nanjing, China).

For co-IP assays, 1 × 10^7^ cells were lysed in NETN buffer on ice for 30 min. The lysates were then incubated with 30 μL of conjugated S-beads (for SFB-tagged pull-down assay) (MilliporeSigma, Burlington, MA, USA) for 2 h at 4 °C. The immunoprecipitates were washed with lysis buffer three times before immunoblot analysis. The following primary antibodies were used: rabbit anti-YTHDF2 [1:1000, 71283S, Cell Signaling Technology (CST), RRID: AB_3068618], anti-Myc (1:5000, A00704, GenScript), and anti-Flag (1:5000, B3111, Sigma-Aldrich, RRID: AB_2910145). The following secondary antibodies were used: goat anti-mouse immunoglobulin G (IgG) antibody (H&L) [horseradish peroxidase (HRP)] (A00160, GenScript) and goat anti-rabbit IgG antibody (H&L) (HRP) (A00178, GenScript).

### 2.6. Quantitative Real-Time PCR

Total RNA was isolated from cells using the Super FastPure Cell RNA Isolation Kit (Vazyme, Nanjing, China), and cDNA synthesis was performed using 1 μg of total RNA with HiScript IV All-in-One Ultra RT SuperMix for qPCR (Vazyme). The mRNA levels of the specific genes were quantified by SYBR Green qPCR according to the manufacturer’s guidance on a qTOWER3G qPCR System (Analytik Jena, Jena, Germany). The relative mRNA levels were determined using the comparative Ct method with actin as the reference gene, following the formula 2−ΔΔCt. The primers used are listed as follows: *ISG15*, 5′- TGGACAAATGCGACGAACCTC-3′ (forward) and 5′-TCAGCCGTACCTCGTAGGTG-3′ (reverse); *IFIT1*, 5′-AGAAGCAGGCAATCACAGAAAA-3′ (forward) and 5′-CTGAAACCGACCATAGTGGAAAT-3′ (reverse); *MX1*, 5′-GGTGGTCCCCAGTAATGTGG-3′ (forward) and 5′-CGTCAAGATTCCGATGGTCCT-3′ (reverse); *IL-6*, 5′-CCTGAACCTTCCAAAGATGGC-3′ (forward) and 5′-TTCACCAGGCAAGTCTCCTCA-3′ (reverse); *IL1β*, 5′-CCACAGACCTTCCAGGAGAATG-3′ (forward) and 5′-GTGCAGTTCAGTGATCGTACAGG-3′ (reverse); *GAPDH*, 5′-GTCTCCTCTGACTTCAACAGCG-3′ (forward) and 5′-ACCACCCTGTTGCTGTAGCCAA-3′ (reverse), *RPLP0*, 5′-GCAGCATCTACAACCCTGAAG-3′ (forward), CACTGGCAACATTGCGGAC(reverse); *HPRT1*, 5′-CCTGGCGTCGTGATTAGTGAT-3′ (forward), 5′-AGACGTTCAGTCCTGTCCATAA-3′ (reverse).

### 2.7. SARS-CoV-2 Pseudovirus Production and Infection

SARS-CoV-2 (WT) pseudoviruses were packaged and used to infect cells as previously described. Briefly, HEK293T cells were cultured in cell culture dishes and co-transfected with pNL 4.3-luc and DB3.1 SARS-CoV-2 S or Omicron S plasmids, which were kindly provided by Peihui Wang (Key Laboratory for Experimental Teratology of the Ministry of Education and Advanced Medical Research Institute, Cheeloo College of Medicine, Shandong University, Ji’nan, Shandong, China) using VigoFect DNA transfection reagents (Vigorous, Beijing, China) according to the manufacturer’s instructions. The supernatants were collected at 48 and 72 h post-transfection, mixed with polyethylene glycol overnight, then filtered with a 0.45-μm filter, centrifuged at 500× *g* for 5 min, aliquoted, and stored at −80 °C.

For WT SARS-CoV-2 pseudovirus infection, H1299 cells were seeded in 96-well plates at a density of 1 × 10^5^ cells per well and cultured for 12 h. The culture medium was then replaced with a pseudovirus mixture consisting of 100 μL serum-free DMEM and 100 μL pseudovirus supernatant, and the cells were incubated with the pseudovirus mixture for 24 h. After infection, the medium was replaced with complete medium, and the cells were further cultured for 36 h. Luciferase reporter activity was then measured using the Luciferase Assay System kit (Promega, Madison, WI, USA). Briefly, the culture medium was removed, and the cells were rinsed with 1× PBS for 10 min. After removing as much residual wash buffer as possible, 25 μL of 1× lysis reagent was added to each well and incubated for 30 min. Finally, 20 μL of cell lysate was mixed with 100 μL of Luciferase Assay Reagent, and luminescence was measured using a luminometer according to the manufacturer’s instructions and instrument settings.

### 2.8. Authentic Virus Infection

Authentic SARS-CoV-2 infection experiments were performed in a Biosafety Level 3 (BSL-3) laboratory at Fudan University in accordance with national biosafety regulations. The SARS-CoV-2 strain hCoV-19/China/WIV04/2019 was obtained from the Wuhan Institute of Virology. H1299 (WT, YTHDF2-OE, YTHDF2-KO) cells were seeded in 96-well plates at a density of 1 × 10^5^ cells per well and infected with SARS-CoV-2 at an MOI of 2.0. After 1 h of adsorption at 37 °C, the viral inoculum was removed, and cells were washed three times with PBS and cultured in fresh complete medium. Cell supernatants and lysates were collected at 48 h post-infection (hpi).

### 2.9. RNA Immunoprecipitation (RIP) Assay

RIP assays for m^6^A enrichment or YTHDF2 enrichment were performed using the PureBinding^®^ RNA Immunoprecipitation Kit (Geneseed, Guangzhou, China). Briefly, 2 × 10^7^ cells were lysed in 1 mL of Buffer A supplemented with protease inhibitor and RNase inhibitor, followed by incubation on a rotator at 4 °C for 30 min. The lysates were clarified by centrifugation at 10,000× *g* for 15 min at 4 °C, and a portion of the supernatant was reserved as the input control. Protein A/G beads were pretreated with Buffer A and cell lysed, and then incubated with 5 μg of the indicated antibody or control IgG at 4 °C for 2 h to form antibody-bead complexes. The complexes were then incubated with the cleared lysates at 4 °C overnight with rotation. After washing 3–5 times with Buffer B or Buffer C, the immunoprecipitated complexes were collected. RNA was subsequently eluted and purified using the columns provided in the kit. The antibodies used were IgG (Cell Signaling Technology, Danvers, MA, USA), YTHDF2 (Cell Signaling Technology) and N6-Methyladenosine (m6A) (Cell Signaling Technology). The co-precipitated RNAs were analyzed by RT–qPCR.

### 2.10. Immunofluorescence

For immunofluorescence staining, cells were seeded onto coverslips in 6-well plates and cultured for 24 h. The medium was then discarded, and the cells were gently washed three times with 1× PBS for a total of 15 min. Subsequently, the cells were fixed with 4% paraformaldehyde (PFA) (Beyotime) at room temperature for 15 min, followed by another three washes with 1× PBS for 15 min in total. The fixed cells were then permeabilized with 0.5% Triton X-100 (Sigma, Burlington, MA, USA) for 5 min at room temperature. After PBS washing, the samples were blocked with 5% BSA (Sigma) in PBS for 1 h and then incubated with the indicated primary antibodies overnight at 4 °C. On the following day, the cells were washed three times with PBS and incubated with fluorescence-conjugated goat anti-rabbit or goat anti-mouse IgG secondary antibodies (1:1000, Abcam, Cambridge, UK) for 1 h at room temperature. Nuclear staining was performed using 4′,6-diamidino-2-phenylindole (DAPI; Sigma-Aldrich, St. Louis, MO, USA). The coverslips were mounted with FluorSave™ Reagent (EMD Millipore, Burlington, MA, USA), and fluorescence images were captured using an Olympus FV3000 Microscope Imaging System (Olympus, Tokyo, Japan).

### 2.11. Statistical Analysis

All experiments were performed at least three times independently, and data are presented as the mean ± standard error of the mean (SEM) or mean ± standard deviation (SD) as indicated in the figure legends. Statistical analyses were performed using GraphPad Prism 10.0 software (GraphPad Software, San Diego, CA, USA). Differences between two groups were analyzed using an unpaired two-tailed Student’s *t*-test. Multiple group comparisons were performed using one-way analysis of variance (ANOVA) followed by Tukey’s post hoc test, or two-way ANOVA followed by Sidak’s post hoc test for experiments with two independent variables. A *p* value < 0.05 was considered statistically significant (* *p* < 0.05, ** *p* < 0.01, *** *p* < 0.001; ns *p* > 0.05).

## 3. Results

### 3.1. YTHDF2 Is a Core Host Interactor of SARS-CoV-2 RNA

**Figure 1:**
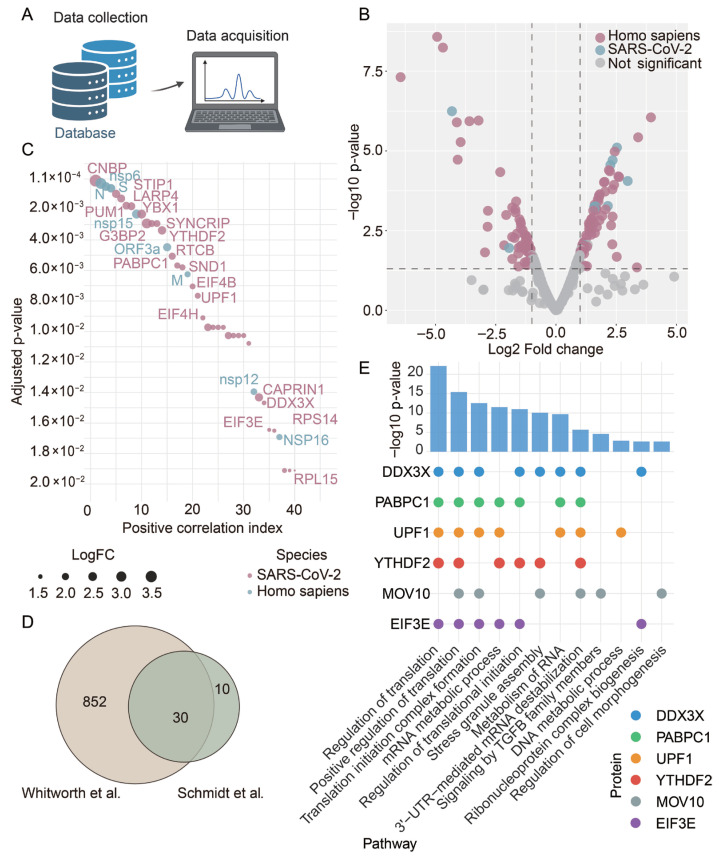
YTHDF2 is a critical interactor of SARS-CoV-2 RNA. (**A**) Schematic of data collection and acquisition. (**B**) Volcano plot of proteome abundance measurements in SARS-CoV-2 RNA and ribonuclease mitochondrial RNA processing (RMRP) RNA of SARS-CoV-2-infected cells. No-significant: −Log_10_ *p* value > 0.05. SARS-CoV-2-encoded proteins are shown in blue; human SARS-CoV-2 RNA interactome proteins are shown in red; no significant proteins are shown in grey. (**C**) The positive correlation analysis of SARS-CoV-2 RNA interactome proteins. Dot sizes scale to the value of LogFC. The horizontal axis represents the ranking of proteins positively associated with the virus. Adjusted *p* value: two-tailed moderated t-test. (**D**) Comparison of SARS-CoV-2 RNA–protein interactome studies in terms of lists of host RBPs identified as associated with viral RNA. The Venn diagrams represent the number of identified proteins common and distinct among the two datasets. [26,27] (**E**) Integrated pathway enrichment and protein-level association analysis. The bar plot displays the statistical significance of pathway enrichment, represented as −log10(*p*-value). The circle plot maps the contribution of six top proteins—DDX3X (blue), PABPC1 (green), UPF1 (orange), YTHDF2 (red), MOV10 (gray), and EIF3E (purple)—to each enriched pathway.

To identify host cellular regulators that mediate SARS-CoV-2 infection and immune evasion, we analyzed the RNA-proteome datasets from Schmidt et al. [26] downloaded from the Mass Spectrometry Interactive Virtual Environment (MassIVE) database (dataset ID: MSV000085734; accessed on 15 August 2023) (Figure 1A). Volcano plot analysis revealed several host proteins significantly enriched in SARS-CoV-2-infected cells, suggesting that they may serve as core viral interactors (Figure 1B). Positive correlation analysis of these SARS-CoV-2 RNA-interacting proteins further identified the top 40 proteins associated with viral load (Figure 1C). To validate these findings, we integrated the dataset with a previously published SARS-CoV-2 RNA interactome dataset [27], and identified 30 overlapping host proteins (Figure 1D), confirming these factors as conserved viral RNA interactors across independent studies.

Gene Ontology (GO) enrichment analysis of the 30 overlapping proteins was performed using the Metascape database (https://metascape.org/gp/#/main/step1; accessed on 8 December 2024) revealed significant enrichment for biological processes related to translation regulation, mRNA metabolic process, and metabolism of RNA (Figure 1E), highlighting the importance of host RNA regulatory machinery in SARS-CoV-2 infection. Six proteins—DDX3X, PABPC1, UPF1, YTHDF2, MOV10, and EIF3E—were found to be central to these enriched pathways (Figure 1E). Among these, YTHDF2 was of particular interest due to its well-characterized role as an m^6^A reader in RNA decay and the established involvement of the m^6^A pathway in viral pathogenesis [13,25]. Together, these data demonstrate that YTHDF2 is a potential key host factor in SARS-CoV-2 infection, warranting further functional characterization.

### 3.2. YTHDF2 Promotes SARS-CoV-2 Infection and Replication

**Figure 2:**
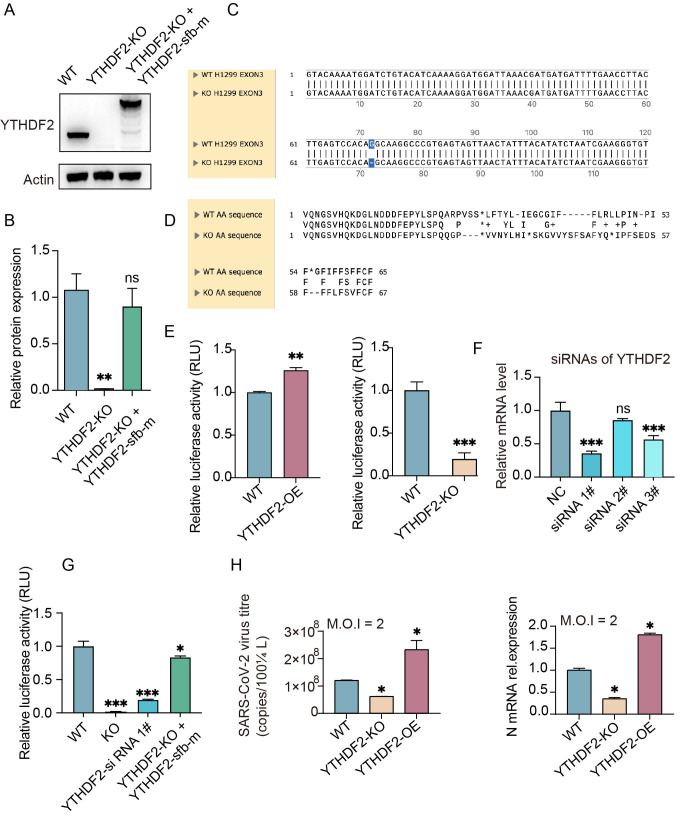
YTHDF2 promotes pseudovirus infection and authentic SARS-CoV-2 replication in H1299 cells. (**A**) Validation of WT, YTHDF2 knockout (YTHDF2-KO) and YTHDF2 rescue cells. YTHDF2-KO H1299 cells were generated using the CRISPR-Cas9 system. YTHDF2-rescue H1299 cells were established by transfection with a cSFB-tagged YTHDF2-m plasmid, which was engineered to evade CRISPR-Cas9-mediated cleavage. The YTHDF2 protein expression was confirmed by Western blotting with an anti-YTHDF2 antibody. (**B**) Quantification of the Western blot results shown in panel (**A**). YTHDF2 protein levels were quantified from three independent experiments. (**C**,**D**) Comparison of the DNA and predicted protein sequences between WT and YTHDF2-KO cells. (**E**) Luciferase assays were performed to evaluate the effect of YTHDF2 on SARS-CoV-2 pseudovirus infection in H1299 cells. WT, YTHDF2-OE, and YTHDF2-KO H1299 cells were infected with luciferase-expressing SARS-CoV-2 pseudovirus for the indicated times, followed by measurement and normalization of luciferase activity. YTHDF2-OE H1299 cell lines were established using cSFB-tagged YTHDF2 plasmids transfection, single clone selection. (**F**) Relative YTHDF2 mRNA levels in NC- and YTHDF2-siRNA-transfected cells. Total RNA was collected 48 h after siRNA transfection and analyzed by RT-qPCR. (**G**) Luciferase assays evaluating pseudovirus infection efficiency in WT, YTHDF2-KO, YTHDF2-siRNA, and YTHDF2-rescue H1299 cells. (**H**) YTHDF2-KO H1299 or YTHDF2-OE cells were infected with SARS-CoV-2 (MOI = 2), and SARS-CoV-2 progeny titers were measured in the cell supernatants by RT-qPCR assay at 48 h post infection. Expression of the SARS-CoV-2 N gene was examined in the cells at 48 h post infection by RT-qPCR. Data are mean ± SD. *n* = 3 independent experiments. One-way ANOVA followed by Tukey’s test. Different lowercase letters represent significant differences between groups (*p* < 0.05). ns *p* > 0.05; * *p* < 0.05, ** *p* < 0.01; *** *p* < 0.001.

YTHDF2 is known to mediate the degradation of m^6^A-modified mRNAs [21]. To explore the functional role of YTHDF2 during SARS-CoV-2 infection, we first established stable H1299 cell lines with YTHDF2 overexpression (YTHDF2-OE) or YTHDF2 knockout (YTHDF2-KO), given that H1299 cells are susceptible to SARS-CoV-2 pseudovirus infection [28]. Western blotting verified ablation of YTHDF2 in YTHDF2-KO cells, and the YTHDF2 rescue validation (Figure 2A,B). Furthermore, DNA sequencing and predicted amino acid sequence analysis verified the CRISPR-Cas9-mediated editing of the YTHDF2 gene (Figure 2C,D).

We next used a SARS-CoV-2 pseudovirus system to assess the effect of YTHDF2 on viral entry. Luciferase reporter assays showed that YTHDF2 overexpression increased pseudovirus infection efficiency, while YTHDF2 knockout reduced infection efficiency (Figure 2E). In addition, we designed siRNAs targeting YTHDF2 and performed pseudovirus infection assays (Figure 2F,G). Consistently, YTHDF2 knockdown also reduced infection efficiency, while re-expression of YTHDF2 restored infection efficiency.

To confirm these findings with authentic virus, we infected YTHDF2-OE and YTHDF2-KO cells with SARS-CoV-2 (MOI = 2.0) and quantified viral replication at 48 hpi. qRT-PCR analysis showed that viral gRNA levels in the supernatant were significantly increased in YTHDF2-OE cells and drastically reduced in YTHDF2-KO cells compared to WT cells (Figure 2H). Consistent with this, viral N expression was significantly upregulated in YTHDF2-OE cells and downregulated in YTHDF2-KO cells (Figure 2H). Collectively, these results demonstrate that YTHDF2 acts as a pro-viral factor that promotes SARS-CoV-2 entry and replication.

### 3.3. The SARS-CoV-2 N mRNA Is the Major m^6^A-Modified Target of YTHDF2

**Figure 3:**
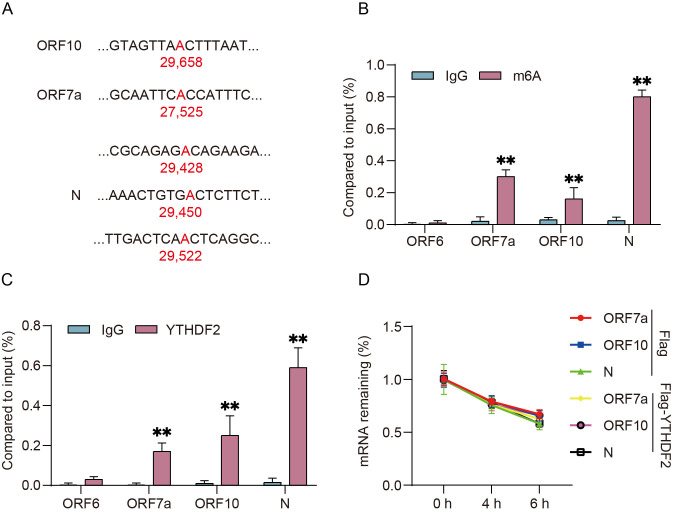
Identification of SARS-CoV-2 N mRNA as the predominant m^6^A-modified viral transcript targeted by YTHDF2. (**A**) Schematic illustration of m^6^A modification sites in SARS-CoV-2 ORF10 (1 site), ORF7a (1 site) and N (3 sites) transcripts. Red “A” indicates m^6^A modification sites. Red colored ‘A’ means the m^6^A modification sites. (**B**) H1299 cells were transfected with indicated vectors for 24 h. m^6^A-IP-qPCR analysis of m^6^A enrichment on the corresponding viral mRNAs. (**C**) H1299 cells were transfected with indicated vectors for 24 h. RIP assay of the association between YTHDF2 and indicated mRNAs was detected using qRT-PCR. (**D**) H1299 cells were co-transfected with ORF7a, ORF10, N and Flag empty vector or Flag-tagged YTHDF2 vector for 24 h and subsequently incubated with 1 μg/mL actinomycin D for the indicated times. ORF7a, ORF10 and N mRNA levels were examined using qRT-PCR. Data are mean ± SD. n = 3 independent experiments. One-way ANOVA followed by Tukey’s test. Different lowercase letters represent significant differences between groups (*p* < 0.05). ** *p* < 0.01.

As a core m^6^A reader protein, YTHDF2 has been widely reported to recognize and regulate target mRNAs in an m^6^A-dependent manner [22]. We hypothesized that YTHDF2 may also participate in the viral life cycle by interacting with SARS-CoV-2 transcripts through m^6^A modifications. We first analyzed the m^6^A modification sites of SARS-CoV-2-encoded transcripts [19,29], and then chose three viral genes with potential m^6^A modification sites: ORF10 (1 site), ORF7a (1 site), and N (3 sites) (Figure 3A). To validate these predictions, we performed m^6^A-IP assays in H1299 cells transfected with individual viral proteins and found that all three viral mRNAs were enriched in the m^6^A-IP fraction, with the N mRNA exhibiting the highest level of m^6^A enrichment (Figure 3B). We next performed RNA immunoprecipitation (RIP) assays and confirmed that YTHDF2 directly binds to ORF10, ORF7a, and N mRNAs, with the strongest binding to the N mRNA (Figure 3C).

To determine whether YTHDF2 binding affects the stability of these viral mRNAs, we treated transfected H1299 cells with actinomycin D (a transcription inhibitor) and quantified viral mRNA levels over time. qRT-PCR analysis showed that YTHDF2 overexpression had no significant effect on the half-life of ORF10, ORF7a, or N mRNAs (Figure 3D), indicating that YTHDF2 does not regulate the stability of these viral transcripts. These results demonstrate that the SARS-CoV-2 N is the most heavily m^6^A-modified viral mRNA and is the primary target of YTHDF2 among SARS-CoV-2 viral RNAs.

### 3.4. N Protein Interacts with and Co-Localizs with YTHDF2

**Figure 4:**
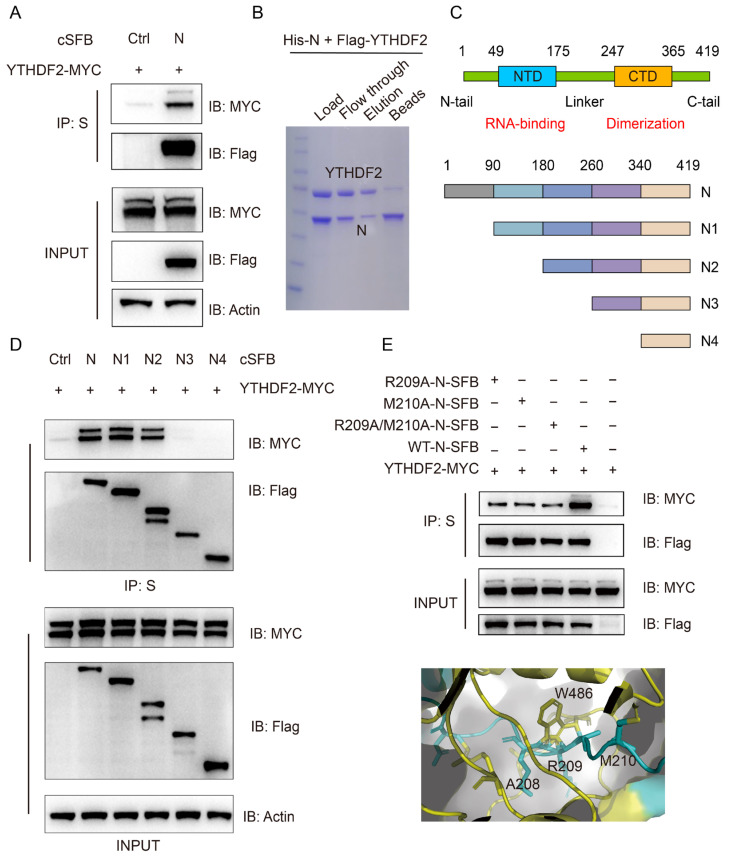
YTHDF2 interacts with N protein. (**A**) Co-immunoprecipitation (Co-IP) analysis of the interaction between YTHDF2 and N protein. HEK293T cells were co-transfected with Myc-tagged YTHDF2 and C-terminal SFB (cSFB)-tagged N protein as indicated. The cell lysates were incubated with S-beads, and immunoprecipitates were analyzed by immunoblotting. (**B**) In vitro binding assay using purified Flag-YTHDF2 and His-N proteins from 293F cells. Purified proteins were incubated with Flag-conjugated beads, and protein complexes were visualized by Coomassie blue staining. (**C**) Schematics showing N protein sequence structure domain deletion mutants. CTD, C-terminal domain; NTD, N-terminal domain. (**D**) Mapping of the YTHDF2-interacting domain in N protein. HEK293T cells were co-transfected with Myc-YTHDF2 and cSFB-tagged wild-type or mutant N constructs. Cell lysates were subjected to Co-IP using S-beads, followed by immunoblotting. Co-IP analysis of the interaction between YTHDF2 and N protein. (**E**) Co-IP analysis of the interaction between YTHDF2 and WT N protein or the R209A, M210A, and R209A/M210A mutants. The protein interaction model was predicted using AlphaFold 3 and visualized with PyMOL (v2.5.7). YTHDF2 is shown in yellow, and the N protein is shown in blue.

Given that YTHDF2 does not degrade viral mRNAs, we next investigated whether YTHDF2 interacts with SARS-CoV-2 viral proteins, particularly the N protein. Co-IP assays in HEK293T cells co-transfected with Myc-tagged YTHDF2 and cSFB-tagged N protein showed that YTHDF2 was specifically co-immunoprecipitated with the N protein (Figure 4A). To confirm this interaction, we performed an in vitro binding assay using purified Flag-tagged YTHDF2 and His-tagged N protein. Coomassie blue staining confirmed that the two purified proteins formed a stable complex in vitro (Figure 4B), demonstrating a direct physical interaction between YTHDF2 and the N protein independent of other cellular factors.

To identify the specific domain of the N protein responsible for binding to YTHDF2, we generated a series of N protein deletion mutants (Figure 4C) and performed Co-IP assays. We found that deletion of the aa 180–260 region completely abrogated the interaction between the N protein and YTHDF2, while deletions of other domains had no significant effect (Figure 4D), indicating that the aa 180–260 region was essential for the N-YTHDF2 interaction. To further identify the key residues involved in this interaction, we used AlphaFold 3 to predict the binding interface between the N protein and YTHDF2. The prediction indicated that residues A208, R209, and M210 of the N protein may contribute to the interaction with W486 of YTHDF2 (Figure 4E). We therefore generated the R209A, M210A, and R209A/M210A mutants and examined their interaction with YTHDF2 by co-IP alongside WT N protein. The results suggested that residues R209 and M210 contribute to the interaction between N protein and YTHDF2, although additional residues may also be involved in mediating this binding (Figure 4E).

**Figure 5:**
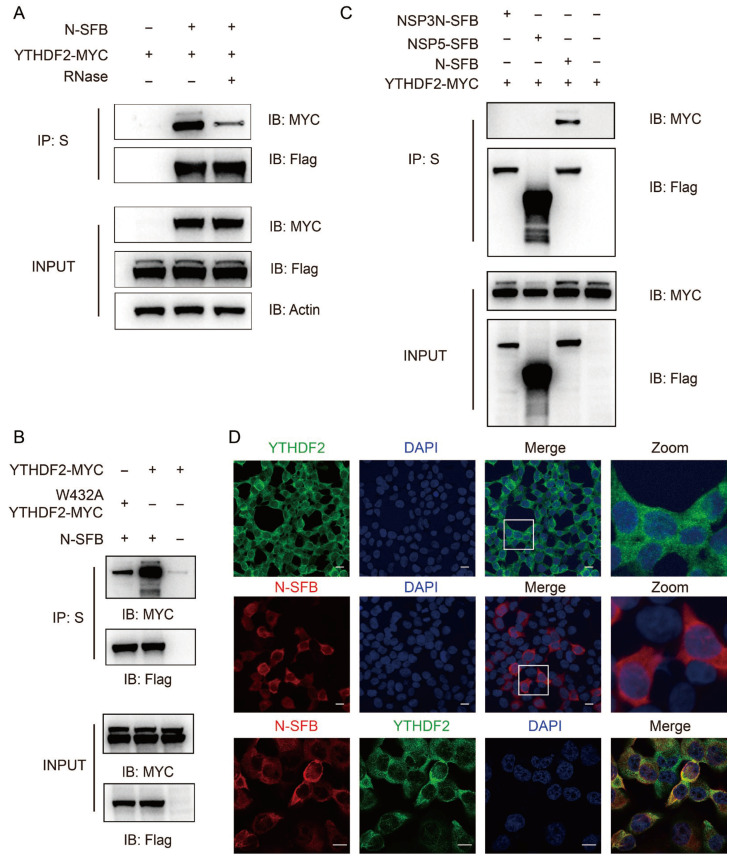
N protein specifically interacts and co-localizes with YTHDF2 in a partially RNA-dependent manner. (**A**) RNase A resistance Co-IP assay. HEK293T cells were co-transfected with Myc-YTHDF2 and cSFB-N. Cell lysates were left untreated or treated with RNase A (1 μg/mL), followed by immunoblotting. (**B**) Co-IP analysis of the interaction between YTHDF2, YTHDF2-W432A and N protein. HEK293T cells were co-transfected with Myc-tagged YTHDF2, YTHDF2-W432A and C-terminal SFB (cSFB)-tagged N protein as indicated. The cell lysates were incubated with S-beads, and immunoprecipitates were analyzed by immunoblotting. (**C**) Co-IP analysis of the interaction between N, NSP3N, NSP5 and YTHDF2 protein. HEK293T cells were co-transfected with Myc-tagged YTHDF2 and C-terminal SFB (cSFB)-tagged N, NSP3N and NSP5 protein as indicated. The cell lysates were incubated with S-beads, and immunoprecipitates were analyzed by immunoblotting. (**D**) Co-localization of YTHDF2 and N protein in HEK293T cells. Immunofluorescence staining was performed using anti-Flag (N, red) and anti-YTHDF2 (green) antibodies; nuclei were stained with DAPI (blue). Scale bars, 20 μm.

To determine whether RNA is required for the interaction between N protein and YTHDF2, we treated cell lysates with RNase A prior to co-immunoprecipitation assays. RNase treatment partially attenuated the N–YTHDF2 interaction (Figure 5A), and the W432A mutation in YTHDF2 likewise partially reduced this binding (Figure 5B), indicating that their association is partially RNA-dependent and may be stabilized by m^6^A-modified RNAs, while the direct protein–protein interaction identified earlier remains intact. To further assess the specificity of this interaction, NSP3N and NSP5 SFB-tagged viral proteins were subjected to co-immunoprecipitation with YTHDF2. Among the proteins result, only N protein interacted with YTHDF2, supporting the specificity of this binding (Figure 5C).

We next performed immunofluorescence staining to examine the subcellular localization of the N protein and YTHDF2. In HEK293T cells transfected with SFB-tagged N protein, the N protein was localized primarily in the cytoplasm, consistent with previous reports [9]. YTHDF2 was also found to be predominantly cytoplasmic, and the two proteins showed extensive co-localization in the cytoplasm (Figure 5D). Collectively, these results demonstrate that the SARS-CoV-2 N protein directly interacts with YTHDF2 and co-localizes with YTHDF2 in the cytoplasm.

### 3.5. N Protein Suppresses Antiviral ISG Expression in a YTHDF2-Dependent Manner

**Figure 6:**
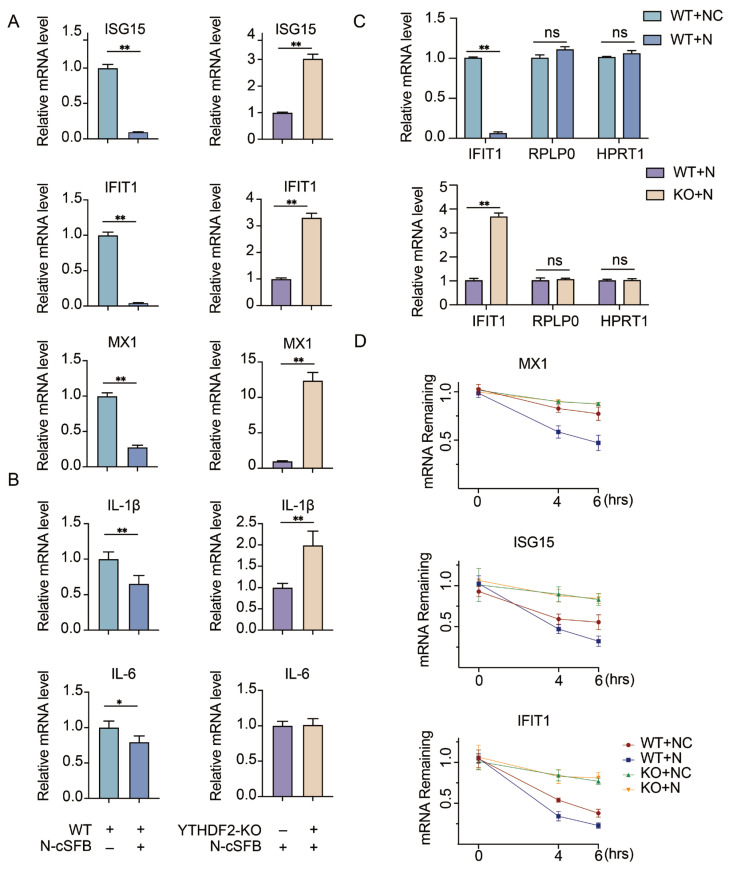
YTHDF2 mediates SARS-CoV-2 N protein-induced suppression of antiviral immune responses. (**A**) qRT-PCR analysis of antiviral ISG mRNA levels (ISG15, IFIT1, MX1) in WT and YTHDF2-KO H1299 cells transfected with control or N-cSFB vector. (**B**) qRT-PCR analysis of pro-inflammatory cytokine mRNA levels (IL-1β, IL-6) in the same groups as in (A). (**C**) qRT-PCR analysis of IFIT1, RPLP0 and HPRT1 in the same groups as in (A). (**D**) WT and YTHDF2-KO H1299 cells were transfected with either empty cSFB vector or cSFB-N for 24 h, followed by stimulation with IFN-α (500 U/mL) for 12 h. Then cells were then treated with 1 μg/mL actinomycin D for the indicated times, and the mRNA levels of MX1, ISG15, and IFIT1 were measured by qRT-PCR. Data are presented as mean ± SEM (n = 3 independent experiments). Statistical significance was analyzed by two-way ANOVA. ns *p* > 0.05, * *p* < 0.05, ** *p* < 0.01.

Given that the SARS-CoV-2 N protein has been reported to antagonize host innate immune responses [10,11], we hypothesized that the N-YTHDF2 interaction may promote the degradation of host antiviral mRNAs to suppress innate immunity. To test this hypothesis, we overexpressed the N protein in wild-type (WT) and YTHDF2-knockout (YTHDF2-KO) H1299 cells, and measured the mRNA levels of representative antiviral interferon-stimulated genes (ISGs: ISG15, IFIT1, MX1) and pro-inflammatory cytokines (IL-6, IL1*β*) by qRT-PCR. In WT H1299 cells, N protein overexpression significantly reduced the transcript levels of ISG15, IFIT1, MX1, IL-6, and IL1*β* (Figure 6A,B), confirming the potent innate immune suppressive function of the N protein. Importantly, the ability of N protein to downregulate these antiviral and pro-inflammatory genes was largely abolished in YTHDF2-KO cells (Figure 6A,B), indicating that the immune inhibitory effect of N protein is largely dependent on YTHDF2.

To determine whether this mRNA degradation is selective, we also measured the transcript levels of RPLP0 (ribosomal protein lateral stalk subunit P0) and HPRT1 (hypoxanthine phosphoribosyltransferase 1), two commonly used housekeeping genes with relatively stable expression that were included as negative controls, and compared them with IFIT1. The results showed that N protein expression affected antiviral transcripts but had no obvious effect on RPLP0 or HPRT1, indicating that the mRNA reduction is selective rather than global (Figure 6C).

Furthermore, we performed an actinomycin D (ActD) chase assay to examine whether the N–YTHDF2 complex accelerates the degradation of host antiviral mRNAs. The results showed that MX1, ISG15, and IFIT1 mRNAs decayed more rapidly in the presence of the N–YTHDF2 complex, supporting the conclusion that N protein cooperates with YTHDF2 to promote the turnover of antiviral transcripts (Figure 6D).

**Figure 7:**
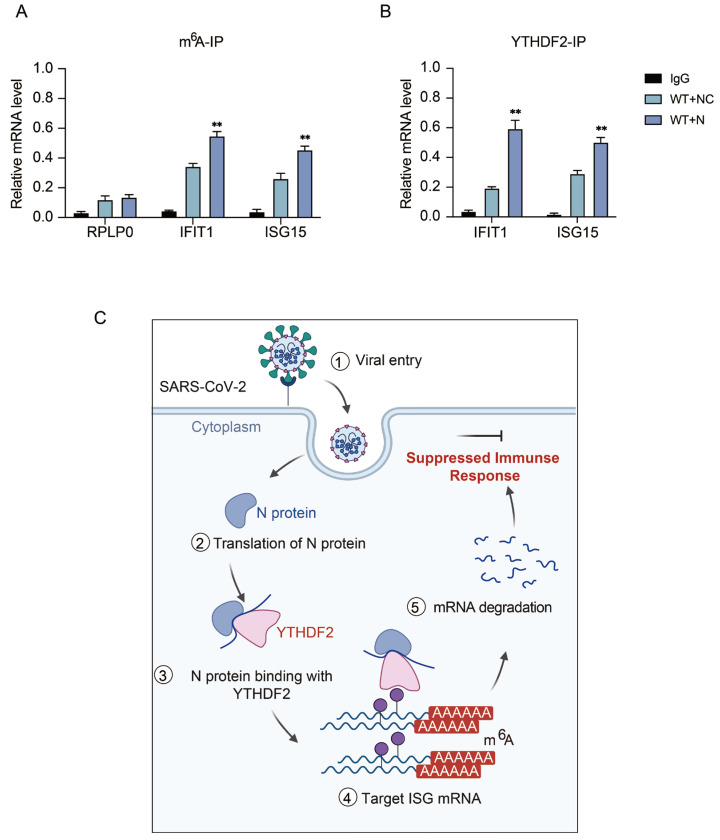
YTHDF2 mediates SARS-CoV-2 N protein-induced suppression of antiviral immune responses. (**A**) H1299 cells were transfected with either empty cSFB vector or cSFB-N for 24 h, followed by pseudovirus infection for 24 h. m^6^A-IP-qPCR analysis of m^6^A enrichment on the RPLP0, IFIT1 and ISG15 mRNAs. (**B**) H1299 cells were transfected with either empty cSFB vector or cSFB-N for 24 h, followed by pseudovirus infection for 24 h. YTHDF2-IP-qPCR analysis of YTHDF2 enrichment on the IFIT1 and ISG15 mRNAs. (**C**) Schematic mechanistic model of the study. Created in BioRender. Sui, Y. (2026) https://BioRender.com/yb8l77k ((accessed on 14 April 2026)) Data are presented as mean ± SEM (n = 3 independent experiments). Statistical significance was analyzed by two-way ANOVA. ** *p* < 0.01.

To determine whether the N–YTHDF2 complex accelerates the degradation of host antiviral mRNAs by enhancing YTHDF2 binding to antiviral transcripts, we first performed m^6^A-RIP-qPCR to examine the m^6^A enrichment of representative antiviral mRNAs. The results showed that, in the presence of N protein, the m^6^A enrichment of IFIT1 and ISG15 transcripts was significantly increased, whereas the housekeeping transcript RPLP0 showed no obvious change (Figure 7A). We next performed YTHDF2-RIP-qPCR to assess the association of YTHDF2 with these transcripts. Consistent with the m^6^A-IP results, YTHDF2 binding to IFIT1 and ISG15 mRNAs was markedly enhanced in the presence of N protein compared with WT cells (Figure 7B). These findings suggest that SARS-CoV-2 N protein promotes the recognition and binding of YTHDF2 to m^6^A-modified antiviral transcripts, thereby facilitating their subsequent degradation.

Collectively, these results support a novel mechanistic model (Figure 7C) in which the SARS-CoV-2 N protein hijacks the host m^6^A reader YTHDF2 to selectively degrade antiviral ISG and pro-inflammatory cytokine mRNAs, thereby dampening the host innate immune response and facilitating viral immune evasion.

## 4. Discussion

The m^6^A RNA methylation pathway critically regulates host–virus interactions, representing a molecular tug-of-war in which the host deploys m^6^A machinery to mount antiviral defenses, while viruses have evolved countermeasures to subvert it [12,13]. YTHDF2, a key m^6^A reader with conserved RNA decay activity [21,22], plays context-dependent roles in viral infections, acting as either a pro-viral or antiviral factor [25]. Previous studies have established that YTHDF2 promotes the replication of certain RNA viruses, such as influenza A virus and porcine epidemic diarrhea virus, by suppressing the expression of antiviral interferon-stimulated genes through passive host responses [21,22]. In this study, we reveal that SARS-CoV-2 employs a distinct strategy. Instead of being passively suppressed by YTHDF2 as seen in other viral contexts, the virus actively hijacks this m^6^A reader through its N protein and redirects it to serve viral interests.

The RNA-proteome dataset analysis identified YTHDF2 as a core host interactor of SARS-CoV-2 RNA. Functional assays confirmed YTHDF2 acts as a pro-viral factor, promoting SARS-CoV-2 pseudovirus entry and authentic viral replication; conversely, YTHDF2 knockout potently inhibits viral infection. Unlike these viruses, YTHDF2 does not regulate SARS-CoV-2 viral RNA stability, including the highly m^6^A-modified N mRNA (Figure 3D). This unexpected observation prompted us to investigate whether YTHDF2 interacts with SARS-CoV-2 viral proteins, ultimately identifying the N protein as its primary viral binding partner.

We demonstrated that the SARS-CoV-2 N protein directly interacts with YTHDF2, and RNase treatment assay showed their interaction is partially RNA-dependent—likely stabilized by m^6^A-modified RNA (viral or cellular). This is consistent with both proteins binding m^6^A-modified RNAs [14,15], suggesting m^6^A-modified RNA may act as a “bridge” to enhance their interaction. However, our in vitro binding assay with purified proteins confirmed a direct protein–protein interaction independent of other cellular factors or RNA, highlighting the complexity of this association.

Critically, the N protein suppresses key antiviral ISGs (ISG15, IFIT1, MX1) and pro-inflammatory cytokines (IL-6, IL1B) in a largely YTHDF2-dependent manner. These results confirm YTHDF2 is an essential mediator of N’s immunosuppressive activity. Given YTHDF2 promotes degradation of m^6^A-modified cellular mRNAs [21,22], we propose the N protein hijacks YTHDF2 to redirect its RNA decay activity toward host antiviral mRNAs, silencing the innate immune response. This mechanism differs from previously reported N protein immune evasion strategies, highlighting its multifunctional role in subverting host immunity.

Notably, the N protein is the most conserved structural protein among coronaviruses [8]. This suggests the N-YTHDF2 interaction axis may be a conserved immune evasion strategy for other coronaviruses (e.g., SARS-CoV, MERS-CoV). Future studies will investigate whether other coronavirus N proteins interact with YTHDF2 and exert similar effects, which could inform broad-spectrum anti-coronavirus therapy development.

In addition, our findings identify the N-YTHDF2 interaction as a promising antiviral target. Given the conservation of N and YTHDF2, such therapeutics may have broad-spectrum activity against coronaviruses and other RNA viruses exploiting the m^6^A pathway for immune evasion.

## 5. Conclusions

This study reveals that SARS-CoV-2 hijacks the host m^6^A reader YTHDF2 to suppress antiviral immunity. We show that the SARS-CoV-2 N protein directly binds YTHDF2 and redirects its RNA decay activity from viral RNA toward host antiviral transcripts including ISG15, IFIT1, and MX1. This immunosuppressive effect is largely YTHDF2-dependent, as it is lost in YTHDF2-knockout cells. These findings uncover a post-transcriptional immune evasion mechanism wherein a viral protein usurps an m^6^A reader to silence antiviral gene expression, establishing the N-YTHDF2 axis as a promising therapeutic target.

## Data Availability

The data presented in this study are available on request from the corresponding author.

## Abbreviations

The following abbreviations are used in this manuscript:

ANOVA	Analysis of Variance
BSL-3	Biosafety Level 3
Co-IP	Co-Immunoprecipitation
COVID-19	Coronavirus Disease 2019
DAPI	4′,6-Diamidino-2-Phenylindole
DMEM	Dulbecco’s Modified Eagle’s Medium
ECL	Enhanced Chemiluminescence
FBS	Fetal Bovine Serum
GO	Gene Ontology
hpi	Post-Infection
IFN	Interferon
IF	Immunofluorescence
ISG	Interferon-Stimulated Gene
IAV	Influenza A Virus
m^6^A	N^6^-Methyladenosine
MOI	Multiplicity of Infection
mRNA	Messenger RNA
N	Nucleocapsid
ORF	Open Reading Frame
PEDV	Porcine Epidemic Diarrhea Virus
PEI	Polyethyleneimine
PVDF	Polyvinylidene Difluoride
qRT-PCR	Quantitative Real-Time PCR
RIP	RNA Immunoprecipitation
RNP	Ribonucleoprotein
SARS-CoV-2	Severe Acute Respiratory Syndrome Coronavirus 2
SDS-PAGE	Sodium Dodecyl Sulfate-Polyacrylamide Gel Electrophoresis
SEM	Standard Error of the Mean
SD	Standard Deviation
VOCs	Variants of Concern
WT	Wild-Type
YTHDF2	YTH Domain Family Protein 2
KO	Knockout
OE	Overexpression
